# Rheology as a monitoring tool for the biochemical parameters of insect cell-baculovirus system

**DOI:** 10.1007/s10529-026-03796-4

**Published:** 2026-10-05

**Authors:** Milena Miyu Teruya, Júlia Públio Rabello, Fernanda Angela Correia Barrence, Thais Oliveira Mendes da Silva, Nicole Cristina Sarmento, Douglas Amaral dos Santos, Noemi Nosomi Taniwaki, Eutimio Gustavo Fernández Núñez

**Affiliations:** 1 Laboratório de Engenharia de Bioprocessos. Escola de Artes, Ciências e Humanidades (EACH), Universidade de São Paulo, Rua Arlindo Béttio, 1000, São Paulo, SP CEP 03828-000 Brazil; 2 Anton Paar Brasil, Rua José de Magalhães, 646, Vila Clementino, São Paulo, SP CEP 04026-090 Brazil; 3 Núcleo de Microscopia Eletrônica, Centro de Procedimentos Interdisciplinares, Instituto Adolfo Lutz, Avenida Dr. Arnaldo, 355, Pacaembu, São Paulo, SP CEP 01246-000 Brazil

**Keywords:** Rheology, VLP bioprocess, Empirical modeling, AIC, Main effects ANOVA

## Abstract

**Purpose:**

Process Analytical Technology (PAT) tools are increasingly applied to improve monitoring of biopharmaceutical processes; however, few techniques have explored their use in liquid bioprocesses based on insect cell cultures. This work evaluated whether rheological parameters could be explored as monitoring indicators for biochemical variables during virus-like particle (VLP) production using the insect cell–baculovirus expression system.

**Methods:**

Batch bioreactor cultivations of *Spodoptera frugiperda* (Sf9) cells were conducted under non-infected conditions and during rabies and SARS-CoV-2 VLP production. Rheological properties were determined by steady-shear cone-plate rheometry and correlated with viable and total cell densities, extracellular protein concentration, and viral titer. Empirical models were generated and ranked using Akaike’s Information Criterion, and predictive performance was assessed by root mean square error. Statistical significance was evaluated using analysis of variance.

**Results:**

All cultures exhibited non-Newtonian, pseudoplastic behavior, with decreases in the flow behavior index associated with increasing cell density and protein concentration. Exponential functions were most frequently selected among the best-ranked empirical models, followed by rational, hyperbolic, logarithmic, and power equations. No statistically significant effect of rheological parameter or infection condition on prediction accuracy was observed (p > 0.05).

**Conclusion:**

Rheological properties reflected compositional changes occurring during insect cell cultivations; however, under the conditions studied, rheology did not significantly improve quantitative monitoring of key biochemical parameters using other approaches. These findings indicate that while rheology may provide complementary process information, further investigation is required before its application as a robust PAT tool for VLP production.

**Supplementary Information:**

The online version contains supplementary material available at https://doi.org/10.1007/s10529-026-03796-4.

## Introduction

The upstream stage for different biopharmaceutical products can be monitored through various approaches, such as Raman and Infrared spectroscopy techniques, providing accurate measurements of critical parameters throughout the product generation length (Shaikh et al. 2018). These kinds of spectroscopy techniques are part of Process Analytical Technology (PAT) tools, which allow for verifying the bioprocess during its early development, seeking a better understanding, and later establishing automatic control loops useful at the commercial scale to ensure final product quality and successful process performance (Ramachandran 2017; Shaikh et al. 2018). Nevertheless, the complexity and singularities of these processes open an opportunity to keep improving and introducing new methods to monitor some biochemical parameters over bioreactions (Pavurala et al. 2025).

In this context, the rheological properties of microbial or cell culture could be employed (Simões et al. 2020), seeking a better understanding of bioconversion progress and associated biochemical changes along the process, as these characteristics, such as shear stress, could impact cell growth and expression (Wyma et al. 2018). And, as rheology describes the fluid’s response to applied forces, it allows mainly the classification of a liquid sample as Newtonian or non-Newtonian depending on whether the viscosity remains constant with respect to the shear rate (Ibarz and Barbosa-Cánovas 2003). Thus, rheological parameters, which depend on the sample’s chemical composition and the morphology of biocatalysts (e.g., host cells and viruses), could be used to control and monitor the quality of the final product and complex mixtures throughout the process (Amanullah et al. 2003).

However, rheological assay methodologies vary to suit the study, mainly because each material responds differently to a defined set of experimental conditions; thus, some parameters may be more critical for characterizing the sample. Additionally, precautions are important when performing rheological assays to minimize experimental noise, including maintaining consistent sample-preparation steps, determining the geometry, preconditioning before the assay begins (e.g., achieving the specified temperature and spreading the sample homogeneously across the geometry surface), and loading an adequate sample volume (Suman 2025). Nonetheless, to date, as these authors note, there is no established method in the literature for monitoring biochemical data alongside these rheological parameters when seeking to produce a biopharmaceutical product with insect cell cultures, as most of the studies in this area corresponds to solids production (Simões et al. 2020), biological material analysis (e.g. blood samples), mechanical force and metabolite concentration rheology influence in plant, bacteria or mammalian cell cultures (Wyma et al. 2018). And rheological studies within bioreactor assays frequently aimed to evaluate the impact of these properties on polymer production (Fernandes et al. 1991) or even optimize sludge treatment, seeking to decrease the energy needed to improve filtration capacity, mostly influenced by the wastewater viscosity (Laera et al. 2007), and the membrane life-span decrease due to biofouling within these processes (Patsios et al. 2015).

Therefore, a pharmaceutical process that could benefit from different monitoring strategies is the production of virus-like particles (VLP). There are already some vaccines developed with recombinant protein products obtained through this system, generally using the insect cell-baculovirus expression vector system. Nevertheless, improved process characterization could facilitate faster production development and higher productivity (Contreras‐Gómez et al. 2014). As some process characteristics may affect VLP productivity when using this platform, such as cell growth kinetics, which correlates to optimal infection time, cell concentration at infection, and multiplicity of infection (MOI) (Radford et al. 1997; Huang et al. 2025), closely related to viral titer during infection (Contreras‐Gómez et al. 2014), which can be assessed using these PAT techniques.

Because the rheological characterization of insect cell culture or baculovirus infection for producing virus-like particles is not commonly correlated with biochemical parameters, to the best of these authors’ knowledge, there is no model in the literature for this analysis. Therefore, this work aims to assess rheological parameters as a monitoring tool for insect cell cultures with and without virus infection, using rabies and SARS-CoV-2 VLP production as a biopharmaceutical upstream model. This goal was achieved by correlating rheological parameters with biochemical data, focusing on viable and total cell density, total extracellular protein, and viral titer. Furthermore, the study assessed whether these rheological parameters, as well as infection or non-infection conditions, could impact the accuracy of biochemical parameter predictions.

## Materials and methods

### Biochemical data acquisition

#### Cell lines and culture media

*Spodoptera frugiperda* (*Sf9*) insect cells (ATCC1711) were cultured in EX-CELL^®^ CD Insect Cell Medium (Catalog n° 24381 C, Sigma-Aldrich, Missouri, USA) in 100 mL Schott flasks (Schott AG™, Rheinland-Pfalz, Germany) with 20 mL working volume and an initial cell density of 1 × 10^6^ cells/mL. And *Sf9* Easy Titer (*Sf9* ET) cell line, kindly given by Professor Dr. Ralph Hopkins and Dr. Dominic Esposito from the Protein Expression Laboratory located at the National Cancer Institute of Frederick (Maryland, USA), was cultured similarly using Sf-900™ III SFM (Catalog n° 12,658,027, Gibco™, Massachusetts, USA) medium supplemented with 2.5% fetal bovine serum (HI FBS, Catalog n° 10,082–147, Gibco™, Massachusetts, USA) for viral titer assays. Cultures were agitated at 110 rpm using a Celltron (Infors HT, Bottmingen, Switzerland) shaker at 28 °C in a PHCbi Cell Culture Incubator (PHC Group, Tokyo, Japan).

#### Rabies and SARS-CoV-2 recombinant baculoviruses

Previous studies were used to generate (Bernardino et al. 2021; Moura Dias et al. 2025) and propagate (Oliveira Guardalini et al. 2023; Guardalini et al. 2024; de Oliveira Guardalini et al. 2025) viral stock of both rabies (carrying rabies glycoprotein and matrix protein genes-RBV-R-G and RBV-R-M, respectively) and SARS-CoV-2 (carrying spike and membrane protein-RBV-S-S and RBV-S-M, respectively) recombinant baculoviruses (RBV). The shaker speed was maintained at 110 rpm to obtain viral stocks in parallel with cell cultivation for bioreactor inoculum, using the same equipment described in the previous section.

#### Bioreactor experiments

Three assays were performed using a Minifors 2 bioreactor (vessel volume 1.5 L; Infors HT, Bottmingen, Switzerland) connected to a computer running eve^®^ (Infors HT, Bottmingen, Switzerland) software. All these runs were performed at an impeller rotation of 80 rpm, a gas flow rate of 0.1 volume of gas per volume of liquid per minute at 30% air saturation, a temperature of 28 °C, and an initial viable cell density of approximately 5 × 10^5^ cells/mL.

The first batch was performed with a working volume of 500 mL, without infection, collecting 14 samples throughout the assay, which lasted around 160 h. The second batch was performed using 400 mL as working volume, with infection with RBV-S-S MOI 3 and RBV-S-M MOI 2 when cell density achieved around 1 × 10^6^ cells/mL. Twelve samples were collected during approximately 125 h of the experiment. And for the third batch, working volume was settled at 400 mL, with infection with RBV-R-G MOI 3 and RBV-R-M MOI 2, when viable cell density achieved nearly 4 × 10^6^ cells/mL, infection parameters studied and determined in previous work (data not shown). 14 samples were collected during approximately 150 h of assay.

All samples were separated for cell counting and viability assay, rheological experiments, viral titer, and total protein quantification. Samples were clarified through centrifugation at 500 × *g* for 5 min for rheological analysis, and stored at 4 °C or − 80 °C for later viral titration and Bradford assays, respectively. At the end of each run, infected batches were harvested to obtain product for transmission electron microscopy and further analysis (not in focus in this work), thus the bioreactor suspension was clarified through 500 × *g* for 20 min and stored at −80 °C.

#### Cell density and viability, viral titration, and total protein quantification

Cell density and viability were determined following a previously established protocol (Aragão Tejo Dias et al. 2025b). Viral titration was assessed using *Sf9* ET cells, as described earlier (Consoni Bernardino et al. 2025). Pierce™ Bradford Plus Protein Assays Kit (Catalog n° 23,236, Thermo Scientific™, Massachusetts, USA) was used for total protein quantification, following the manufacturer’s instructions.

#### Characterization of rabies and SARS-CoV-2 VLP through transmission electron microscopy (TEM)

Bioreactor product for VLP production (45 mL) was ultracentrifuged at 80,000 × *g* for 3 h (Optima™ MAX-XP Ultracentrifuge, MLA-50 rotor, Beckman Coulter, Indiana, USA), the pellet was resuspended overnight with 500 µL PBS 1x (Catalog n° 70,011,044, Gibco™, USA) and later, 100 µL of each sample was applied separately to a carbon film supported copper grids (Grid size 300 mesh, Catalog n° 931,519, Sigma-Aldrich, Germany) to incubate for 30 min. Grids were washed 3 times with PBS for 10 min then kept for 1 h with a mixture of 20 µL of each primary antibody (Anti-Rabies Glycoprotein–1:100, LifeSpan BioSciences, USA –, and Anti-Rabies Matrix protein–1:100, Cusabio, China –, for rabies VLP grids; Anti-SARS-CoV-2 Spike protein–1:100, Catalog n° MA535946, Thermo Fisher, USA –, and Anti-SARS-CoV-2 Membrane protein–1:100, Catalog n° PA1-41,160, Thermo Fisher, USA –, for SARS-CoV-2 VLP grids), homogenized every 15 min. Grids were washed again 3 times with PBS and incubated with a mixture with 50 µL of each colloidal gold-conjugated secondary antibody (Anti-Mouse–1:50, 10 nm colloidal gold, Catalog n° A31561, Thermo Fisher, USA –, for Anti-Rabies Glycoprotein and Anti-SARS-CoV-2 Spike protein; and Anti-Rabbit–1:50, 5 nm colloidal gold, Catalog n° A31565, Thermo Fisher, USA –, for Anti-Rabies Matrix and Anti-SARS-CoV-2 Membrane proteins), resuspended each 15 min. Grids were then contrasted with 2% uranyl acetate for 1 min, and images were acquired using a JEOL transmission electron microscope (model JEM-1011, Massachusetts, USA).

### Rheology parameters modeling

#### Rheological data acquisition

Two samples with cells and another two clarified, 600 µL each, were separated for rheology assays and used only once for rheological analysis throughout the batches. These were analyzed with Rheometer MCR 92 (Anton Paar GmbH, Graz, Austria) using a cone-plate geometry (Anton Paar GmbH, Graz, Austria), as this requires a lower sample volume and allows for a steady shear rate across the geometry surface (Suman 2025). The equipment was connected to a computer with software RheoCompass version 1.34 (Anton Paar GmbH, Graz, Austria) for shear rate (γ, 1/s), shear stress (τ, Pa), apparent viscosity (*µ*_*app,*_ mPa · s), and torque (mN · m) acquisition. Equipment temperature was settled at 28 °C, the same temperature for bioreactor runs, and 21 points during a shear rate ramp from 0.1 to 100 1/s were analyzed for each sample. During the measurements, the room was maintained with no airflow or movement around the equipment.

#### Data modeling and ranking

Data collected from bioreactor monitoring, biochemical (viable cell density–X_v_, total cell density–X_t_, extracellular protein concentration–EP, viral titer–VT) and rheological (shear stress, shear rate, apparent viscosity) parameters, were organized and used for analyses in Excel spreadsheets (Office Home 2024, Microsoft Corporation, Washington, USA) (Supplementary material Table 1–3). X_v_ and X_t_ from infected batches, as these present different behaviors before and after infection, were analyzed separately during modeling. EP was correlated to bioreactor rheology using data from the entire batch, even for infected assays. Although baculovirus infection in determined phase of cell cycle arrests its machinery and inhibits protein expression (Contreras‐Gómez et al. 2014), this process is not immediate, host cell proteins expression declines over 18 h post infection (Du and Thiem 1997), and cells could also be lysed in 3–5 days post-infection (Kost et al. 2005), releasing other proteins to the extracellular media, thus all these proteins are quantified at once when assessed through Bradford assay, data used for modeling. Rheological data obtained were correlated to X_v_, X_t,_ and EP. And for VT, the analysis was made using only post-infection data from both infected batches, analyzed with rheological data from clarified samples.

Using the power-law model (Eq. 1) (Wang et al. 2013) with observed data for γ and τ, it was possible to determine the consistency index (*k*) and flow behavior index (*n*) for each point collected for each sample. The average of *k*, *n,* and *µ*_*app*_ of the two samples with or without cells was calculated to use for modeling with each biochemical parameter.

1 $$\tau =k\times {\gamma }^{n}$$

Then, the Rank Model tool from OriginProTrial Version 2026 (OriginLab Corporation, Maryland, USA) was used to correlate biochemical (Y) and rheological (X) data using empirical models available within this tool default configuration, considering Exponential, Growth/Sigmoidal, Hyperbola, Logarithm, Peak Functions, Polynomial, Power, Rational, Waveform, PFW (a second category to organize peak functions for data fitting) and Baseline functions categories.

To rank the calibrated models, Akaike’s Information Criterion (AIC) (Eq. 2) (OriginLab) was used to determine the best prediction model for each correspondence (Chakrabarti and Ghosh 2011). The model with the smallest AIC was selected for each biochemical vs. rheological correlation, as the lowest value would represent the model whose estimated values were the most approximate to the uncertain data behavior, considering the models generated. This criterion also considers the amount of data and parameters, as these could create noise and result in under- or overfitting (Burnham and Anderson 2010).

$$\mathrm{A}\mathrm{I}\mathrm{C}=Nln\left(\frac{RSS}{N}\right)+2K, when \frac{N}{K}\ge 40$$

2 $$\mathrm{A}\mathrm{I}\mathrm{C}=Nln\left(\frac{RSS}{N}\right)+2K+ \frac{2K\left(K+1\right)}{N-K-1}, when \frac{N}{K}<40$$

where N is the sample size, RSS is the residual sum of squares (Eq. 3) (OriginLab), and K is the number of estimable parameters in an approximating model.

3 $$\mathrm{R}\mathrm{S}\mathrm{S}=\sum_{i=1}^{N}{\left({y}_{i}-{\widehat{y}}_{i}\right)}^{2}$$

where ${y}_{i}$ is the observed value and ${\widehat{y}}_{i}$ is the fitted value.

Also, for the best model selected through AIC, root mean square error (RMSE) was also calculated (Eq. 4) (Moura Dias et al. 2024), as it is a criterion commonly used to assess model quality, where smaller values indicate better data fit (Amangeldi et al. 2021; Ahmed et al. 2022).

4 $$RMSE=\sqrt{\frac{\sum_{i=1}^{N}{\left({y}_{i}-{\widehat{y}}_{i}\right)}^{2}}{N}}$$

#### Statistical analysis

Main effects analysis of variance (ANOVA) was performed to determine whether rheological parameter (*k*, *n,* or *µ*_*app*_), and bioreaction condition (with or without infection) are statistically significant, with 95% confidence interval, to monitor a certain biochemical parameter, and if so, which is the best variable or parameter seeking to allow biochemical monitoring along the bioprocess. These analyses were performed in software Statistica^®^ version 14.0.1 (TIBCO, California, USA).

As a bioreactor condition, looking to verify whether infection was significant, pre-infection data from the three assays, while modeling each biological with rheological parameter pair were united to generate only one RMSE, with Eq. 4, for each comparison. Similarly, data derived from infected batches, post-infection phase, were also compiled, using Eq. 4. These RMSE values for pre- and post-infection phases were indicated as RMSE” to distinct from RMSE calculated and used to select the best model. Thus, rheological parameter and bioreaction condition were used as independent variables, and RMSE” were used as a dependent variable. However, for viral titration, which bioreactor condition data was separated only in infected with SARS-CoV-2 RBV’s or rabies RBV’s, a One-Way ANOVA was performed to assess if a rheological parameter would impact this biochemical parameter monitoring. Also, if indicated statistical significance in any analyses, a Tukey’s HSD test post hoc was also performed within the same software tool.

## Results and discussion

### Biochemical and rheological parameters modeling.

Rheological assessment of the flow behavior index, n, using samples with cells across entire batches (Supplementary material Table 1–3) to represent bioreactor cultures showed that all assays, except for one sample from SARS-CoV-2 RBV infection, exhibited shear-thinning pseudoplastic behavior, as values were below 1 (Alizadeh and Pal 2025). With most non-Newtonian responses according to the increase of total cell density and protein concentration, similar to data presented in a study with microalgae concentrates (Attar et al. 2022), a previous work concerning bacteria cultured to produce polymers (Blunt et al. 2019), a research with plant-based foods proteins (Badjona et al. 2025), and production of polysaccharides using fungus culture (Meng et al. 2023), where higher cell concentration, for cultures studies, or higher product concentration were related to increased pseudoplastic behavior. The non-infected batch presented a higher consistency index, *k,* as cell density and protein concentration increased, similarly to previous works (Pamboukian and Facciotti 2005; Li et al. 2020). Viscosity may be affected by cell concentration, but it is not the only parameter, and could also vary depending on DNA, protein, and other extracellular components (Newton et al. 2016), as well as metabolite formation (Wyma et al. 2018), as verified during all the assays, where viscosity varies, but not following cell densities, protein, or viral titer values. As for infected batches, considering the viral titer correlation with clarified samples, both assays’ infected samples also exhibited non-Newtonian, pseudoplastic behavior, as observed in the study of bacteriophage encapsulation (Silva Batalha et al. 2021), but this behavior did not correspond with viscosity as indicated when analyzing alongside with cell density or protein concentration. However, these VLP production bioreactor samples did not present an increase or decrease pattern for any other rheological parameters.

With these biochemical and rheological data prepared, using the Rank Model tool within Origin software, one model, with the lowest AIC value, was selected for each correlation (Tables1–3). Nonetheless, some models that presented lower values for AIC indicated modeling error, possibly due to sample size that could lead to over-parameterization (OriginLab), even with functions with minimum parameters needed. However, despite the different possible functions to empirically model these relations, for all the models, only exponential, power, rational, logarithm and hyperbole functions were selected, as indicated by the category related to the functions used. Exponential functions were the most prevalent equation used to correlate biochemical and rheological parameters; likewise, an exponential model was used to correlate fungus culture density with *k* and *n* (Schmidell et al. 2001).

**Table 1:** Best models for each biochemical variable ($\mathrm{y}$) correlated to the consistency index ($\mathrm{x}=\mathrm{k}$)

Biochemical variable	Experimental condition	Model category	Degrees of Freedom	Equation	Parameter	Value ± Standard Error	AIC	RMSE
Xv (cells/mL)	No infection	Hyperbola	6	$\mathrm{y}=\mathrm{a}\frac{\mathrm{b}\mathrm{x}}{1+\mathrm{b}\mathrm{x}}$	a	−3,468,378.483 ± 1,739,693.776	232.038	939,114.309
					b	−62.965 ± 11.833		
	Exponential growth (SARS-CoV-2 VLP production)	Exponential	3	$\mathrm{y}={\mathrm{e}}^{\mathrm{x}-\mathrm{A}}$	A	−13.375 ± 0.174	113.441	194,675.439
	Exponential growth (rabies VLP production)	Exponential	4	$\mathrm{y}={\mathrm{e}}^{\mathrm{x}-\mathrm{A}}$	A	−13.856 ± 0.226	140.712	475,047.985
	Post-infection phase (SARS-CoV-2 VLP production)	Exponential	4	$\mathrm{y}={\mathrm{e}}^{\mathrm{x}-\mathrm{A}}$	A	−13.208 ± 0.157	130.565	172,045.183
	Post-infection phase (rabies VLP production)	Exponential	6	$\mathrm{y}={\mathrm{e}}^{\mathrm{x}-\mathrm{A}}$	A	−15.186 ± 0.115	202.008	1,120,326.368
Xt (cells/mL)	No infection	Exponential	6	$\mathrm{y}=\mathrm{a}\left(1{-\mathrm{e}}^{\mathrm{b}\mathrm{x}}\right)$	a	−899,498.947 ± 718,224.088	232.971	995,196.802
					b	−208.594 ± 73.143		
	Exponential growth (SARS-CoV-2 VLP production)	Exponential	3	$\mathrm{y}={\mathrm{e}}^{\mathrm{x}-\mathrm{A}}$	A	−13.424 ± 0.173	113.796	202,211.430
	Exponential growth (rabies VLP production)	Exponential	4	$\mathrm{y}={\mathrm{e}}^{\mathrm{x}-\mathrm{A}}$	A	−13.923 ± 0.216	140.934	485,726.630
	Post-infection phase (SARS-CoV-2 VLP production)	Exponential	4	$\mathrm{y}={\mathrm{e}}^{\mathrm{x}-\mathrm{A}}$	A	−13.690 ± 0.020	115.073	36,619.278
	Post-infection phase (rabies VLP production)	Exponential	6	$\mathrm{y}={\mathrm{e}}^{\mathrm{x}-\mathrm{A}}$	A	−15.371 ± 0.088	200.862	1,032,276.007
EP (mg/mL)	No infection	Exponential	7	$\mathrm{y}=1-{\mathrm{e}}^{-\mathrm{A}\mathrm{x}}$	A	17.662 ± 2.344	−47.878	0.034
	Whole batch (SARS-CoV-2 VLP production)	Power	7	$\mathrm{y}={\mathrm{x}}^{\mathrm{A}}$	A	0.443 ± 0.028	−44.947	0.040
	Whole batch (rabies VLP production)	Rational	11	$\mathrm{y}=\frac{1}{\mathrm{x}+\mathrm{A}}$	A	6.985 ± 1.302	−52.915	0.062
VT (PFU/mL)	Post-infection phase (SARS-CoV-2 VLP production)	Power	3	$\mathrm{y}={\mathrm{x}}^{\mathrm{A}}$	A	−2.737 ± 0.056	137.926	4,158,837.257
	Post-infection phase (rabies VLP production)	Power	4	$\mathrm{y}={\mathrm{x}}^{\mathrm{A}}$	A	−2.530 ± 0.052	147.452	932,079.998

**Table 2:** Best models for each biochemical variable ($\mathrm{y}$) correlated to the flow behavior index ($\mathrm{x}=\mathrm{n}$)

Biochemical variable	Experimental condition	Model category	Degrees of freedom	Equation	Parameter	Value ± Standard Error	AIC	RMSE
Xv (cells/mL)	No infection	Exponential	6	$\mathrm{y}={\mathrm{e}}^{\mathrm{a}+\mathrm{b}\mathrm{x}}$	a	17.112 ± 0.353	230.62928	860,340.615
					b	−5.622 ± 1.081		
	Exponential growth (SARS-CoV-2 VLP production)	Exponential	3	$\mathrm{y}={\mathrm{e}}^{\mathrm{x}-\mathrm{A}}$	A	−12.503 ± 0.293	117.0489	305,708.809
	Exponential growth (rabies VLP production)	Exponential	4	$\mathrm{y}={\mathrm{e}}^{\mathrm{x}-\mathrm{A}}$	A	−13.420 ± 0.265	142.00305	540,529.867
	Post-infection phase (SARS-CoV-2 VLP production)	Exponential	4	$\mathrm{y}={\mathrm{e}}^{\mathrm{x}-\mathrm{A}}$	A	−12.849 ± 0.131	128.94087	146,242.910
	Post-infection phase (rabies VLP production)	Exponential	6	$\mathrm{y}={\mathrm{e}}^{\mathrm{x}-\mathrm{A}}$	A	−14.687 ± 0.133	203.8101	1,274,244.546
Xt (cells/mL)	No infection	Exponential	6	$\mathrm{y}={\mathrm{e}}^{\mathrm{a}+\mathrm{b}\mathrm{x}}$	a	17.149 ± 0.360	231.70664	919,561.974
					b	−5.589 ± 1.098		
	Exponential growth (SARS-CoV-2 VLP production)	Exponential	3	$\mathrm{y}={\mathrm{e}}^{\mathrm{x}-\mathrm{A}}$	A	−12.553 ± 0.292	117.41386	318,639.365
	Exponential growth (rabies VLP production)	Exponential	4	$\mathrm{y}={\mathrm{e}}^{\mathrm{x}-\mathrm{A}}$	A	−13.489 ± 0.253	142.22671	552,755.370
	Post-infection phase (SARS-CoV-2 VLP production)	Exponential	4	$\mathrm{y}={\mathrm{e}}^{\mathrm{x}-\mathrm{A}}$	A	−13.307 ± 0.073	127.62587	128,313.002
	Post-infection phase (rabies VLP production)	Exponential	6	$\mathrm{y}={\mathrm{e}}^{\mathrm{x}-\mathrm{A}}$	A	−14.871 ± 0.110	203.70653	1,264,853.060
EP (mg/mL)	No infection	Exponential	7	$\mathrm{y}={\mathrm{B}}^{\mathrm{x}}$	B	0.003 ± 0.001	−44.93612	0.040
	Whole batch (SARS-CoV-2 VLP production)	Logarithm	6	$\mathrm{y}=\mathrm{l}\mathrm{n}\left(\mathrm{a}+\mathrm{b}\mathrm{x}\right)$	a	1.218 ± 0.040	−43.1333	0.032
					b	−0.220 ± 0.075		
	Whole batch (rabies VLP production)	Rational	8	${\mathrm{y}} = \frac{{{\mathrm{a}} + {\mathrm{bx}}}}{{1 + {\mathrm{cx}} + {\mathrm{dx}}^{2} }}$	a	0.028 ±0.020	−56.15844	0.038
					b	−0.093 ± 0.044		
					c	−4.279 ± 0.143		
					d	3.934 ± 0.301		
VT (PFU/mL)	Post-infection phase (SARS-CoV-2 VLP production)	Exponential	3	$\mathrm{y}={\mathrm{B}}^{\mathrm{x}}$	B	1.854 × 10^9^ ± 6.551 × 10^8^	136.74177	3,586,620.157
	Post-infection phase (rabies VLP production)	Exponential	4	$\mathrm{y}={\mathrm{e}}^{\mathrm{x}-\mathrm{A}}$	A	−13.680 ± 0.315	147.88251	973,108.235

**Table 3:** Best models for each biochemical variable (y) correlated to apparent viscosity (${x=\mu }_{app}$)

Biochemical variable	Experimental condition	Model category	Degrees of Freedom	Equation	Parameter	Value ± Standard Error	AIC	RMSE
Xv (cells/mL)	No infection	Exponential	8	$\mathrm{y}={\mathrm{B}}^{\mathrm{x}}$	B	1.441 × 10^7^ ± 3,067,769.346	260.952	1,417,430.174
	Exponential growth (SARS-CoV-2 VLP production)	Exponential	3	$\mathrm{y}={\mathrm{e}}^{\mathrm{x}-\mathrm{A}}$	A	−12.488 ± 0.184	113.840	204,636.128
	Exponential growth (rabies VLP production)	Exponential	4	$\mathrm{y}={\mathrm{e}}^{-\mathrm{A}\mathrm{x}}$	A	−15.857 ± 0.270	141.106	494,135.788
	Post-infection phase (SARS-CoV-2 VLP production)	Exponential	4	$\mathrm{y}={\mathrm{e}}^{-\mathrm{A}\mathrm{x}}$	A	−15.072 ± 0.162	129.729	158,397.201
	Post-infection phase (rabies VLP production)	Exponential	6	$\mathrm{y}={\mathrm{e}}^{\mathrm{x}-\mathrm{A}}$	A	−14.313 ± 0.117	202.176	1,133,882.081
Xt (cells/mL)	No infection	Power	7	$\mathrm{y}=\mathrm{a}{\left(1+\mathrm{x}\right)}^{\mathrm{b}}$	a	2.585 × 10^–9^ ± 3.589 × 10^–8^	264.572	1,326,748.465
					b	53.869 ± 21.402		
	Exponential growth (SARS-CoV-2 VLP production)	Exponential	3	$\mathrm{y}={\mathrm{e}}^{\mathrm{x}-\mathrm{A}}$	A	−12.537 ± 0.183	114.196	212,601.929
	Exponential growth (rabies VLP production)	Exponential	4	$\mathrm{y}={\mathrm{e}}^{-\mathrm{A}\mathrm{x}}$	A	−15.923 ± 0.270	141.701	524,449.858
	Post-infection phase (SARS-CoV-2 VLP production)	Polynomial	3	$\mathrm{y}=\mathrm{A}+\mathrm{B}\mathrm{x}$	A	1,422,636.669 ± 689,548.344	134.492	34,579.539
					B	−615,633.41914 ± 794,871.716		
	Post-infection phase (rabies VLP production)	Rational	5	$\mathrm{y}=\frac{1}{\mathrm{a}+\mathrm{b}\mathrm{x}}$	a	4.159 × 10^–7^ ± 7.997 × 10^–7^	207.832	1,030,069.778
					b	−2.346 × 10^–7^ ± 9.087 × 10^–7^		
EP (mg/mL)	No infection	Power	8	$\mathrm{y}={\mathrm{x}}^{\mathrm{A}}$	A	22.137 ± 1.358	−54.104	0.035
	Whole batch (SARS-CoV-2 VLP production)	Exponential	7	$\mathrm{y}=-\mathrm{l}\mathrm{n}\left(\mathrm{B}\right){\mathrm{B}}^{\mathrm{x}}$	B	0.015 ± 0.004	−43.023	0.045
	Whole batch (rabies VLP production)	Rational	9	$\mathrm{y}=\frac{\mathrm{b}+\mathrm{c}\mathrm{x}}{1+\mathrm{a}\mathrm{x}}$	a	−1.128 ± 8.242 × 10^–4^	−62.324	0.038
					b	0.120 ± 0.016		
					c	−0.135 ± 0.018		
VT (PFU/mL)	Post-infection phase (SARS-CoV-2 VLP production)	Power	3	$\mathrm{y}={\mathrm{x}}^{\mathrm{A}}$	A	−89.342 ± 2.160	138.845	4,665,304.600
	Post-infection phase (rabies VLP production)	Power	4	$\mathrm{y}={\mathrm{x}}^{\mathrm{A}}$	A	−77.676 ± 3.879	152.200	1,498,521.481

### Statistical analysis

**Fig. 1:**
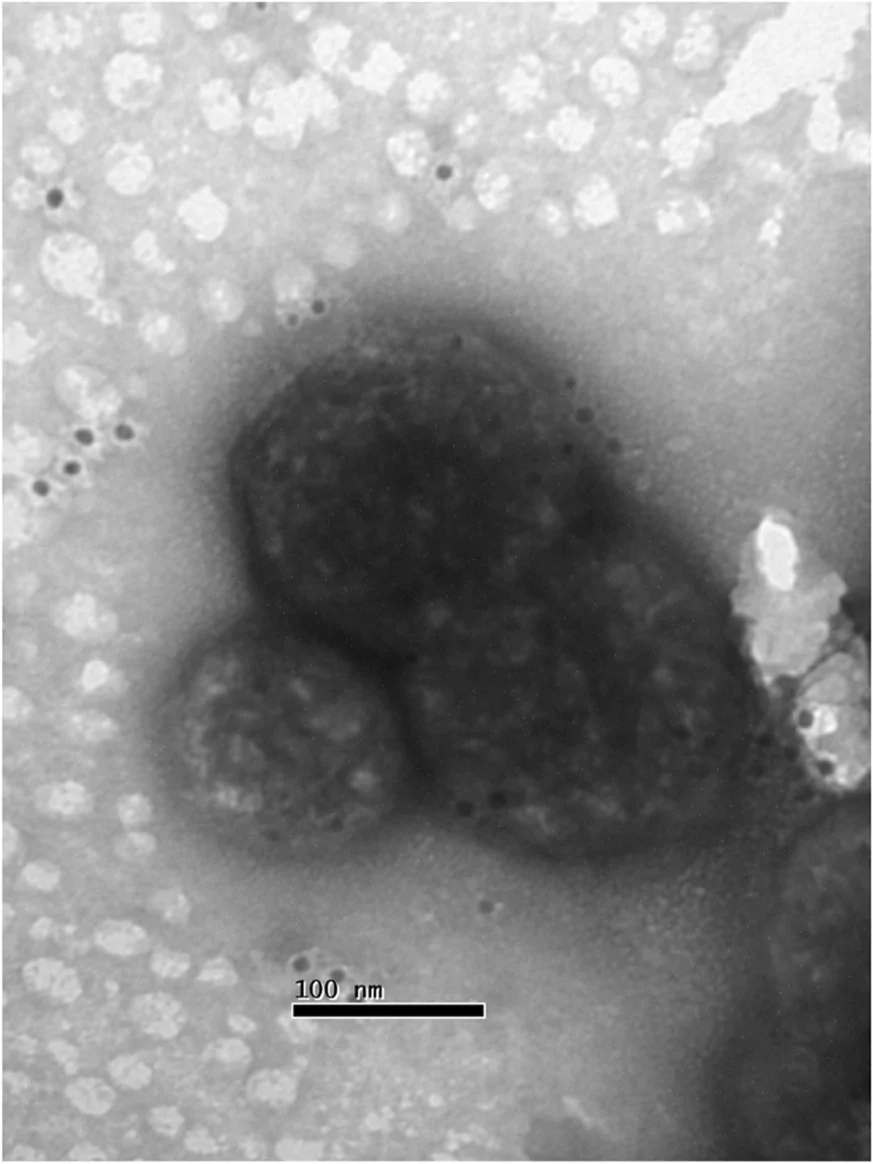
SARS-CoV-2 VLP transmission electron microscopy. Magnification of 200 K presenting particles with diameters of 183.87, 180.80, and 129.42 nm; the average size of all particles detected was 185.72 nm

**Fig. 2:**
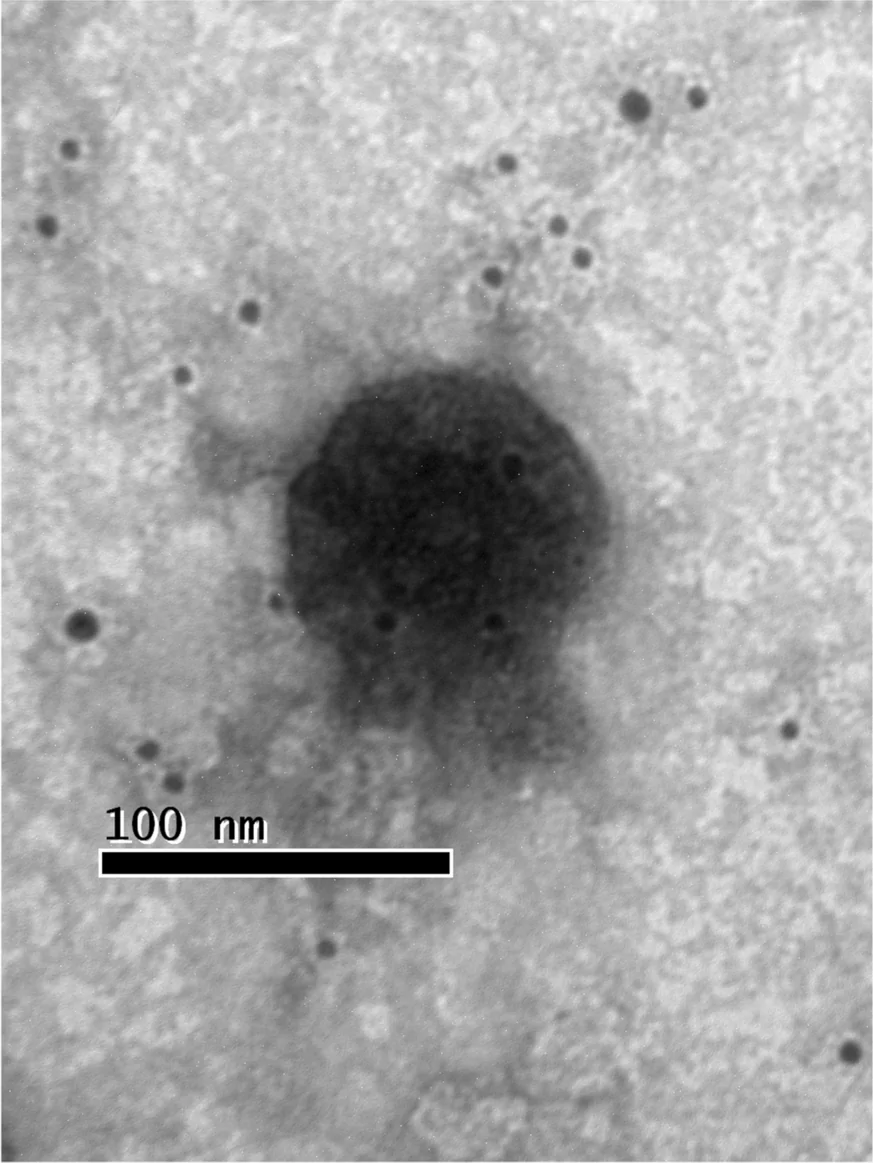
Rabies VLP transmission electron microscopy. Magnification of 200 K presenting particle with 92.91 nm; average size of all particles detected was 107.89 nm

Although different infections were performed to obtain SARS-CoV-2 (Fig. 1) and rabies VLP (Fig. 2) as model for these particle productions, Main Effects ANOVA were done to determine if a rheological variable or an infection process would impact the quality of fit of the models obtained for each biochemical parameter monitored throughout each run. Thus, another RMSE, indicated as RMSE”, was calculated compiling data from the pre-infection stage for the three batches for and from both VLP production assays for post-infection phase. And for viral titer, data for each infected batch was maintained separately for the One-Way ANOVA, and the rheological parameter was the only independent variable.

Among all ANOVA tests, neither rheological parameters nor bioprocess condition presented a significant impact while monitoring viable and total density, total extracellular protein, or viral titer, with p-values > 0.05 (Table 4). Although AIC was used to select the best models, to consider sample size and equation parameters (Burnham and Anderson 2010), models still presented elevated RMSE”, compared to models’ errors when analyzing VLP production with different cell cultures monitored with spectroscopic methods (Moura Dias et al. 2025; Baukmann et al. 2025; Aragão Tejo Dias et al. 2025a), with mathematical adjustment to compare with the error metrics used according to the equations indicated in each paper. Furthermore, these error values could be related to the already indicated difficulty in modeling non-Newtonian fluids (Wyma et al. 2018), or even the sample size available, as some analysis error messages during modeling were due to small database (OriginLab). Thus, suggesting for further studies the necessity of collecting higher number of samples in order to establish a stronger correlation. However, RMSE (Tables 1, 2, and 3) revealed for Xv, Xt, and EP, a prediction capacity within the expected analytical error for quantification techniques of these biochemical variables (Barbosa et al. 2009; Camacho-Fernández et al. 2018). Conversely, VT by rheological parameters in this study showed less accuracy than those reported for viral titration fluorescence assays (Moon et al. 2025), but with RMSE modeling values lower than those presented while monitoring viral particles with Raman spectroscopy (Paiva et al. 2026), corroborating the need for an extended study to provide more data for infection assays in order to establish a better correlation.

**Table 4:** p-values obtained with ANOVA for each biochemical parameter

Biochemical parameter	Rheological parameter	Bioprocess condition
Xv	0.640	0.551
Xt	0.770	0.807
EP	0.645	0.352
VT	0.931	–

The only dependent variable for viral titer (VT) was the rheological parameter.

## Conclusion

The results derived from this work demonstrated that insect cell growth and infection with baculovirus assays to obtain VLP, both pre- and post-infection stages, behave as non-Newtonian fluids with pseudoplastic characteristics, as evidenced by a decreasing flow behavior index. This aligns with other fermentation cultures, which also exhibit behavior in response to increases in cell density. When modeling these rheological and biochemical relations, exponential functions with one parameter were most commonly used, followed by rational, hyperbole, power, and logarithmic functions. Nevertheless, further investigation using a larger dataset could help establish more accurate correlations among these parameters. Besides, analyses showed that rheological parameters or infection had no meaningful impact on all biochemical monitoring, as the RMSE” values were statistically equal.

## Supplementary Information

Below is the link to the electronic supplementary material.

Supplementary file1 (DOCX 43 kb)

## Data Availability

No datasets were generated or analysed during the current study.
