# Supplementary material for: Rheology as a monitoring tool for the biochemical parameters of insect cell-baculovirus system
Source: Biotechnol Lett. 2026 Oct 5;48(5):127. doi: 10.1007/s10529-026-03796-4 (PMC13638685; doi:10.1007/s10529-026-03796-4)
Supplement: Supplementary file 1 [file 10529_2026_3796_MOESM1_ESM.docx]

## Rheology as a monitoring tool for the biochemical parameters of insect cell-baculovirus system

Milena Miyu Teruya^a^, Júlia Públio Rabello^a^, Fernanda Angela Correia Barrence^a^, Thais Oliveira Mendes da Silva^a^, Nicole Cristina Sarmento^a^, Douglas Amaral dos Santos^b^, Noemi Nosomi Taniwaki^c^, Eutimio Gustavo Fernández Núñez^a*^

a Laboratório de Engenharia de Bioprocessos. Escola de Artes, Ciências e Humanidades (EACH), Universidade de São Paulo, Rua Arlindo Béttio, 1000, CEP 03828-000, São Paulo, SP-Brazil
b Anton Paar Brasil, Rua José de Magalhães, 646, Vila Clementino, CEP 04026-090, São Paulo, SP-Brazil
c Núcleo de Microscopia Eletrônica, Centro de Procedimentos Interdisciplinares, Instituto Adolfo Lutz, Avenida Dr. Arnaldo, 355, Pacaembu, CEP 01246-000, São Paulo, SP-Brazil

*Corresponding author: Eutimio Gustavo Fernández Núñez; E-mail: [egfnunez@usp.br](mailto:egfnunez@usp.br), Phone number: +55 (11) 95038-6226

Supplementary material

**Table 1.** Biochemical and rheological parameters used for modeling, data from a non-infected batch. Blank spaces indicate data removed due to negative shear-stress values.

| Sample | Time (h) | Viable cell density (X_v_, cells/mL) | Total cell density (X_T_, cells/mL) | Total protein concentration (EP, mg/mL) | Average consistency index (*k,* Pa.s^n^) (sample with cells) | Average flow behavior index (*n,* dimensionless) (sample with cells) | Average apparent viscosity (*u_app,_,* mPa.s) (sample with cells) | Average consistency index (*k,* Pa.s^n^) (clarified sample) | Average flow behavior index (*n,* dimensionless) (clarified sample) | Average apparent viscosity (*u_app_,,* mPa.s) (clarified sample) |
| --- | --- | --- | --- | --- | --- | --- | --- | --- | --- | --- |
| 1 | 0.0 | 6.000 x 10^5^ | 6.375 x 10^5^ | 0.024 | 2.804 x 10^-3^ | 0.731 | 0.845 | 9.017 x 10^-3^ | 0.328 | 0.836 |
| 2 | 5.5 | 4.208 x 10^5^ | 4.375 x 10^5^ | 0.068 | 5.443 x 10^-3^ | 0.482 | 0.854 | 6.455 x 10^-3^ | 0.344 | 0.895 |
| 3 | 20.3 | 6.375 x 10^5^ | 6.625 x 10^5^ | 0.016 |  |  | 0.864 | 2.901 x 10^-3^ | 0.667 | 0.862 |
| 4 | 28.0 | 7.563 x 10^5^ | 7.750 x 10^5^ | 0.025 | 5.282 x 10^-3^ | 0.503 | 0.885 | 3.929 x 10^-3^ | 0.488 | 0.917 |
| 5 | 45.2 | 1.363 x 10^6^ | 1.438 x 10^6^ | 0.037 |  |  |  | 5.864 x 10^-3^ | 0.466 | 0.894 |
| 6 | 52.0 | 1.433 x 10^6^ | 1.479 x 10^6^ | 0.067 | 4.915 x 10^-3^ | 0.527 | 0.887 | 3.881 x 10^-3^ | 0.560 | 0.854 |
| 7 | 68.3 | 2.219 x 10^6^ | 2.344 x 10^6^ | 0.092 | 5.728 x 10^-3^ | 0.429 | 0.907 |  |  |  |
| 8 | 77.1 | 2.264 x 10^6^ | 2.414 x 10^6^ | 0.124 | 6.037 x 10^-3^ | 0.484 | 0.893 | 7.257 x 10^-3^ | 0.359 | 0.883 |
| 9 | 92.3 | 3.931 x 10^6^ | 4.169 x 10^6^ | 0.144 | 5.651 x 10^-3^ | 0.446 | 0.926 |  |  |  |
| 10 | 100.1 | 4.604 x 10^6^ | 4.817 x 10^6^ | 0.140 |  |  |  | 3.888 x 10^-3^ | 0.605 | 0.872 |
| 11 | 116.3 | 4.058 x 10^6^ | 4.329 x 10^6^ | 0.176 |  |  |  | 2.445 x 10^-3^ | 0.779 | 0.821 |
| 12 | 124.3 | 7.013 x 10^6^ | 7.329 x 10^6^ | 0.196 | 1.064 x 10^-2^ | 0.238 | 0.913 |  |  |  |
| 13 | 139.9 | 1.061 x 10^7^ | 1.131 x 10^7^ | 0.319 |  |  |  | 1.039 x 10^-2^ | 0.224 | 0.846 |
| 14 | 164.0 | 1.040 x 10^7^ | 1.170 x 10^7^ | 0.387 |  |  |  | 7.893 x 10^-3^ | 0.341 | 0.820 |

**Table 2.** Biochemical and rheological parameters used for modeling, data from the infected batch to produce SARS-CoV-2 VLP. Blank spaces correspond to data removed due to negative values for shear stress (data for rheological parameters for samples 2 and 3 although with some negative shear stress values, were maintained to allow analysis). Infection occurred after the 4^th^ sample.

| Sample | Time (h) | Viable cell density (X_v_, cells/mL) | Total cell density (X_T_, cells/mL) | Viral titer (VT, PFU/mL) | Total protein concentration (EP, mg/mL) | Average consistency index (*k,* Pa.s^n^) (sample with cells) | Average flow behavior index (*n,* dimensionless) (sample with cells) | Average apparent viscosity (*u_app,_,* mPa.s) (sample with cells) | Average consistency index (*k,* Pa.s^n^) (clarified sample) | Average flow behavior index (*n,* dimensionless) (clarified sample) | Average apparent viscosity (*u_app_ ,* mPa.s) (clarified sample) |
| --- | --- | --- | --- | --- | --- | --- | --- | --- | --- | --- | --- |
| 1 | 0.0 | 5.250 x 10^5^ | 5.500 x 10^5^ | 0.00 | 0.044 | 2.373 x 10^-3^ | 0.777 | 0.880 | 4.022 x 10^-3^ | 0.657 | 0.831 |
| 2 | 6.5 | 4.500 x 10^5^ | 4.750 x 10^5^ | 0.00 |  | 9.989 x 10^-4^ | 1.042 | 0.886 | 1.169 x 10^-3^ | 1.010 | 0.882 |
| 3 | 19.5 | 6.458 x 10^5^ | 6.792 x 10^5^ | 0.00 | 0.056 | 6.117 x 10^-3^ | 0.747 | 0.926 | 1.028 x 10^-3^ | 1.038 | 0.837 |
| 4 | 27.5 | 9.625 x 10^5^ | 1.006 x 10^6^ | 0.00 | 0.049 | 5.540 x 10^-3^ | 0.506 | 0.851 | 3.892 x 10^-3^ | 0.654 | 0.869 |
| 5 | 43.7 | 7.438 x 10^5^ | 8.250 x 10^5^ | 1.490 x 10^6^ | 0.116 | 3.101 x 10^-3^ | 0.558 | 0.908 | 4.537 x 10^-3^ | 0.512 | 0.899 |
| 6 | 51.5 | 7.000 x 10^5^ | 7.958 x 10^5^ | 3.200 x 10^6^ | 0.112 |  |  |  | 3.032 x 10^-3^ | 0.676 | 0.851 |
| 7 | 67.6 | 6.688 x 10^5^ | 9.219 x 10^5^ | 6.900 x 10^6^ | 0.113 | 1.041 x 10^-2^ | 0.263 | 0.861 |  |  |  |
| 8 | 75.6 | 6.031 x 10^5^ | 8.906 x 10^5^ | 6.900 x 10^6^ | 0.118 | 9.059 x 10^-3^ | 0.343 | 0.855 |  |  |  |
| 9 | 92.0 | 4.750 x 10^5^ | 9.313 x 10^5^ | 3.200 x 10^7^ | 0.163 | 6.325 x 10^-3^ | 0.419 | 0.878 |  |  |  |
| 10 | 99.7 | 4.625 x 10^5^ | 1.000 x 10^6^ | 6.900 x 10^6^ | 0.185 |  |  |  | 7.344 x 10^-3^ | 0.379 | 0.833 |
| 11 | 115.9 | 4.344 x 10^5^ | 9.625 x 10^5^ | 1.490 x 10^7^ | 0.167 |  |  |  | 2.589 x 10^-3^ | 0.774 | 0.838 |
| 12 | 125.2 | 2.563 x 10^5^ | 8.750 x 10^5^ | 3.200 x 10^6^ | 0.186 | 1.135 x 10^-2^ | 0.256 | 0.833 |  |  |  |

**Table 3.** Biochemical and rheological parameters used for modeling, data from the infected batch used to produce rabies VLP. Blank spaces correspond to data removed due to negative values for shear stress. Infection occurred after the 7^th^ sample.

| Sample | Time (h) | Viable cell density (X_v_, cells/mL) | Total cell density (X_T_, cells/mL) | Viral titer (VT, PFU/mL) | Total protein concentration (EP, mg/mL) | Average consistency index (*k,* Pa.s^n^) (sample with cells) | Average flow behavior index (*n,* dimensionless) (sample with cells) | Average apparent viscosity (*u_app_,* mPa.s) (sample with cells) | Average consistency index (*k,* Pa.s^n^) (clarified sample) | Average flow behavior index (*n,* dimensionless) (clarified sample) | Average apparent viscosity (*u_app,_,* mPa.s) (clarified sample) |
| --- | --- | --- | --- | --- | --- | --- | --- | --- | --- | --- | --- |
| 1 | 0 | 5.250 x 10^5^ | 5.750 x 10^5^ | 0 | 0.047 | 6.267 x 10^-3^ | 0.439 | 0.890 | 9.102 x 10^-3^ | 0.338 | 0.865 |
| 2 | 5.7 | 5.406 x 10^5^ | 5.875 x 10^5^ | 0 | 0.059 | 7.474 x 10^-3^ | 0.402 | 0.827 | 4.041 x 10^-3^ | 0.587 | 0.884 |
| 3 | 20.7 | 1.278 x 10^6^ | 1.373 x 10^6^ | 0 | 0.061 | 4.955 x 10^-3^ | 0.535 | 0.868 |  |  |  |
| 4 | 28.2 | 1.109 x 10^6^ | 1.216 x 10^6^ | 0 | 0.120 | 6.845 x 10^-3^ | 0.438 | 0.833 |  |  |  |
| 5 | 44.7 | 1.794 x 10^6^ | 1.856 x 10^6^ | 0 | 0.063 | 1.719 x 10^-2^ | 0.194 | 0.894 |  |  |  |
| 6 | 52.7 | 2.453 x 10^6^ | 2.630 x 10^6^ | 0 | 0.064 |  |  |  | 4.360 x 10^-3^ | 0.564 | 0.874 |
| 7 | 68.7 | 3.797 x 10^6^ | 3.953 x 10^6^ | 0 | 0.112 |  |  |  | 2.898 x 10^-3^ | 0.692 | 0.874 |
| 8 | 77.2 | 3.478 x 10^6^ | 3.647 x 10^6^ | No quantification | 0.131 | 3.348 x 10^-3^ | 0.562 | 0.906 | 4.352 x 10^-3^ | 0.592 | 0.772 |
| 9 | 92.7 | 5.278 x 10^6^ | 5.656 x 10^6^ | 4.060 x 10^5^ | 0.110 | 7.519 x 10^-3^ | 0.401 | 0.860 | 3.398 x 10^-3^ | 0.664 | 0.872 |
| 10 | 100.6 | 4.425 x 10^6^ | 4.713 x 10^6^ | 1.170 x 10^6^ | 0.138 | 5.544 x 10^-3^ | 0.513 | 0.848 | 3.481 x 10^-3^ | 0.599 | 0.884 |
| 11 | 116.9 | 3.753 x 10^6^ | 4.272 x 10^6^ | 3.200 x 10^6^ | 0.183 | 7.119 x 10^-3^ | 0.404 | 0.853 |  |  |  |
| 12 | 124.6 | 5.628 x 10^6^ | 6.831 x 10^6^ | 3.200 x 10^6^ | 0.171 | 6.435 x 10^-3^ | 0.494 | 0.906 | 3.386 x 10^-3^ | 0.652 | 0.863 |
| 13 | 140.5 | 2.603 x 10^6^ | 3.888 x 10^6^ | 2.180 x 10^6^ | 0.307 | 3.426 x 10^-3^ | 0.661 | 0.884 | 3.366 x 10^-3^ | 0.664 | 0.828 |
| 14 | 149.7 | 2.556 x 10^6^ | 4.331 x 10^6^ | 3.200 x 10^5^ | 0.326 | 7.664 x 10^-3^ | 0.347 | 0.885 | 4.990 x 10^-2^ | 0.028 | 10.096 |
